# Influence of Adjustable CeO_2_ Morphology on the Performance of Ambient Hole Transport Layer-Free Carbon-Based Perovskite Solar Cells

**DOI:** 10.1021/acs.energyfuels.5c00518

**Published:** 2025-05-12

**Authors:** Shubhranshu Bhandari, Sreeram Valsalakumar, Mir Sahidul Ali, Tapas K. Mallick, Justin Hinshelwood, Senthilarasu Sundaram

**Affiliations:** † Solar Energy Research Group, Environment and Sustainability Institute, University of Exeter, Penryn Campus, Cornwall TR10 9FE, U.K.; ‡ Department of Polymer Science and Technology, 250209 University of Calcutta, 92 A.P.C Road, Kolkata, West Bengal 700009, India; § Faculty of Environment Science and Economy, University of Exeter, Penryn Campus, Cornwall TR10 9FE, U.K.; ∥ School of Computing, Engineering and Design Technologies, Teesside University, Tees Valley, Middlesbrough TS1 3BX, U.K.

## Abstract

The combined effect of TiO_2_ and CeO_2_ as the electron transport layer (ETL) in the hole transport layer (HTL)-free carbon-based perovskite solar cells (C-PSCs) to enhance performance characteristics is a less explored research area. In this context, we investigated the effect of morphology-tuned CeO_2_ in combination with TiO_2_ in the C-PSCs. Considering the light scattering effect in C-PSCs and the property of extending the light-traveling distance across the photoelectrode, we synthesized rod and cubic CeO_2_ nanostructures. The synthesized nanoparticles were used over the TiO_2_ layer, and their photovoltaic performance was compared to that of the TiO_2_-only C-PSC and analyzed by using impedance and quantum efficiency studies. The light-scattering effect on the C-PSCs, investigated with the diffused reflectance study, found that the rod structure of CeO_2_ provides better light travel toward the photosensitizer, and the highest power conversion efficiency (PCE) of nearly 12.5% was recorded for the rod-shaped CeO_2_ in the HTL-free C-PSC, which is 24% higher compared to a pristine TiO_2_-based C-PSC. Moreover, the devices with rod-shaped CeO_2_ demonstrated suitable charge transport properties along the perovskite layer and a lower charge recombination rate when compared with the cube structure. This work demonstrates a major breakthrough in the performance enhancement of HTL-free C-PSCs by nanomaterial morphology alteration and fabrication engineering, which can significantly influence future research.

## Graphical abstract

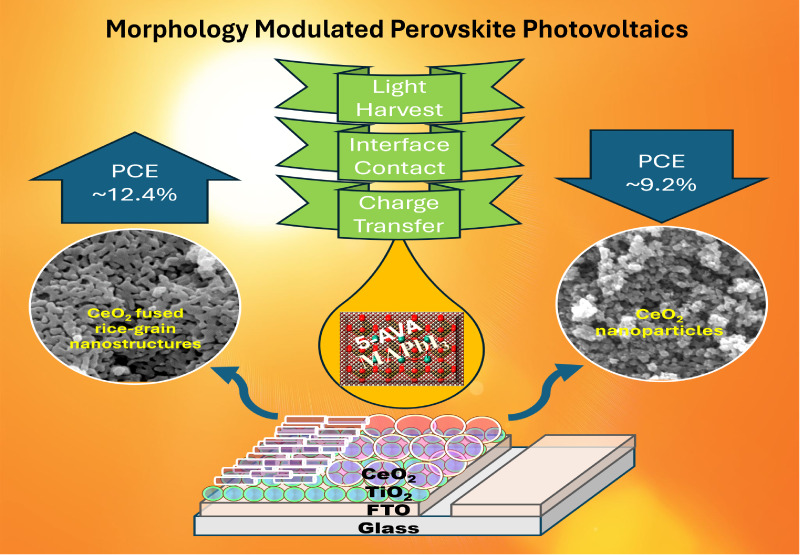

## 1 Introduction

The rapid development of organic–inorganic carbon-based perovskite solar cell (C-PSC) technology over a decade has gained attention from industrial and academic societies.^1–4^ The noteworthy improvement in power conversion efficiency (PCE) from a single digit to over 26% has been observed for traditional noble metal counter electrode devices, which is the same as the single-crystalline silicon solar cells and 1.7% higher than multicrystalline silicon solar cells.^5–10^ However, HTL-free devices are still hovering around a PCE of 15%.^11^ The simple solution-based fabrication method and the unique optoelectronic properties of perovskite nanocrystals and allied layers have attracted scientific curiosity among researchers.^2,8,12–18^ The ETL extracts photogenerated electrons from the absorber or the perovskite and transfers them to the TCO layer.^8,9,15^ Considering the importance of ETL material selection in the fabrication process affects the C-PSC efficiency and stability. A wide range of research studies have been conducted with different ETL materials to address interfacial charge recombination between the ETL and the perovskite layer.^3,5,6,9,15,19–24^

Among the ETL materials, titanium dioxide (TiO_2_) is the most used one in the regular architecture of the C-PSCs.^19,24,25^ Alternatively, other ETL materials, such as ZnO, SnO_2_, BaSnO_3_, CeO_2_, etc., have been utilized in C-PSC fabrication, achieving significant results.^26–28^ The application of different particle sizes and shapes plays a vital role in determining the performance of C-PSCs.^26,29,30^ There have been various studies in terms of the morphology modulation of the ETL and its impact on the PSCs or the C-PSCs.^19,26,29–34^ In 2020, Wu et al. carried out an ETL morphology engineering study to produce TiO_2_ nanocrystals, followed by the doping of Zn into the ETL.^35^ This study demonstrated an average PCE (power conversion efficiency) of 19.87% for Zn-doped nanocrystalline TiO_2_ and 16.95% for nondoped normal TiO_2._ Also, to evaluate the titania macropores in the TiO_2_ layers, in 2021, Khan et al. conducted an extensive study on synthesizing three-dimensional (3-D) hollow anatase TiO_2_ microspheres through the hydrothermal method and assessed their suitability as an effective ETL for the C-PSCs.^36^ In 2020, Wang et al. validated the impact of an amorphous WO_*x*_ (a-WO_*x*_) layer as an interlayer between the perovskite and the TiO_2_ layers.^37^ This study clearly indicated better nonwettability performance of the hybrid ETL (TiO_2_ + a-WO_*x*_) and the improved crystallization of the perovskite layer via enhancing the grain boundary mobility.^37^ The C-PSC with the hybrid ETL showed a PCE of 20.98% and 30 days of stability at room temperature under dark conditions.^37^ In 2022, Bhandari et al. investigated the performance variation of C-PSCs by blending the morphology and the phase of TiO_2_.^38^ This study considered the brookite phase of TiO_2_ and recorded the improved efficiency and effects on charge recombination with respect to different morphologies.^38^ Considering the ETL doping strategies, in 2022, Arshad et al. demonstrated the use of a Ca-doped TiO_2_ layer, which was synthesized through the sol–gel method.^39^ The study indicated the improved performance in the current density, increasing from 15 mA/cm^2^ for the C-PSC with normal TiO_2_ to 19.3 mA/cm^2^ for the Ca-doped layer.^39^ Furthermore, in 2020, Ebrahimi et al. examined the effects of adding the dopant GQDs (graphene quantum dots) in the TiO_2_ layer and indicated that pinhole density was reduced on the perovskite film.^40^ This aided the electron extraction and enhanced the charge mobility in the GQD-doped TiO_2_ layer.^40^ The EIS (electrochemical impedance spectroscopy) analysis from this study indicated a diminished recombination process in the doped layer compared to the normal layer, which provided an improved FF (fill factor) for the C-PSCs.^40^ Li is a widely used doping agent for TiO_2_ layers and several studies have demonstrated performance enhancements due to Li doping.^29,41–45^ In 2020, Teimouri et al. showed increased conductivity and faster electron transport characteristics in the TiO_2_ layer through Li doping.^44^ In this study, capacitance-frequency analysis interpreted higher conductivity and lower trap-state density in the TiO_2_/perovskite layers.^44^ However, device engineering using an electron transport material (ETM) can still play a crucial role, as it influences performance in various ways.

In this aspect, we considered the first of its kind, the coupling of morphology-modulated CeO_2_ nanostructures with TiO_2_ as the combined ETL and conducted performance testing for the ambient HTL-free C-PSC. The C-PSC fabricated with the rod-shaped CeO_2_ particles provided better performance than the standard ETL, and the device with cube-shaped CeO_2_ particles.

## 2 Experimental Section

### 2.1 Synthesis of CeO_2_ Rods and Cubes

The synthesis method was adapted from previous literature with suitable modifications, as shown in the schematic diagram in Figure 1.^46^ To synthesize the CeO_2_ rod particles, cerium nitrate (Ce­(NO_3_))_4_ purchased from Merck was dissolved in deionized (DI) water and rapidly added to 10% NaOH solution and stirred for 10 min at 300 rpm. Afterward, the solution was transferred into an autoclave (130 °C for 24 h), followed by centrifugation and rinsing with water until the pH of the solution reached 7. Finally, the end product from the autoclave was calcined for 5 h at 650 °C.

**Figure 1:**
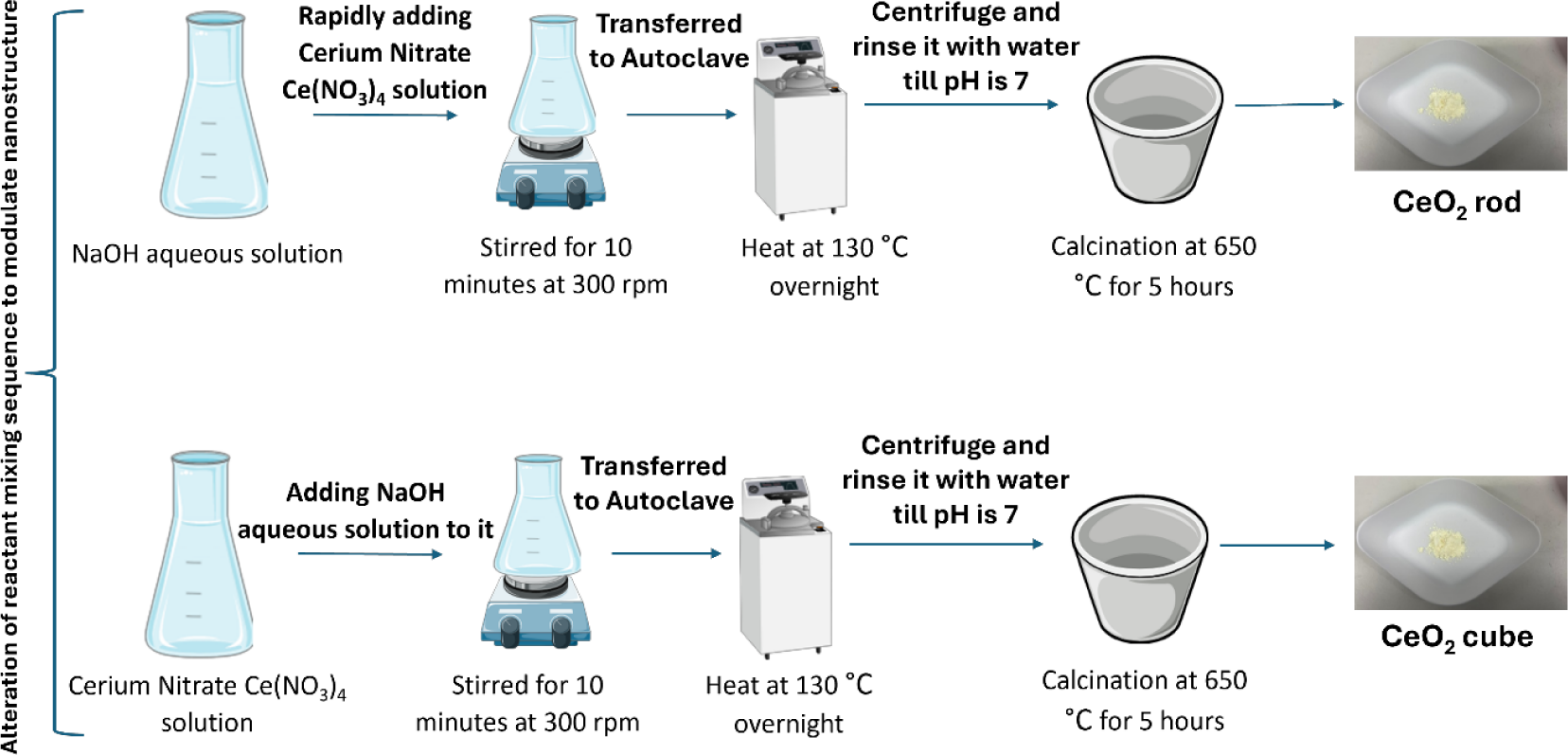
Schematic of CeO_2_ nanostructure syntheses for (a) the rod structure and (b) the cube structure by alteration of reactant mixing.

The alteration of the mixing sequence was followed to synthesize the CeO_2_ cube particles. A 10% NaOH aqueous solution was prepared first. In a separate container, cerium nitrate (Ce­(NO_3_))_4_ was dissolved in DI water, and the initially prepared aqueous NaOH was added to it vigorously with continuous stirring for 10 min at 300 rpm. Then, the solution was transferred to an autoclave (130 °C for 24 h), followed by centrifugation and rinsing with water until the pH reached 7. In the end, similar to the CeO_2_ rod synthesis, the material underwent the calcination process at 650 °C for 5 h.

### 2.2 Device Fabrication

The device fabrication methodology was adopted from previously published articles, and adequate modifications were introduced in this work.^47^ Three batches of C-PSCs with different configurations were fabricated for this study. The C-PSC configuration of each batch is given in Table 1. For the fabrication of all three batches, a 2 cm × 2 cm fluorine-doped tin oxide (FTO) glass substrate with a sheet resistance of 10 Ω sq^–1^ was used. The FTO-coated glass substrate underwent an etching process with diluted HCl and Zn powder. Afterward, the standard cleaning procedures were performed for 20 min each in an ultrasonic bath using detergent, DI water, ethanol, and acetone. Next, an ozone–ultraviolet treatment was performed for 30 min. Subsequent to the standard cleaning procedure, for all three batches, a compact-TiO_2_ (c-TiO_2_) solution was prepared with titanium diisopropoxide bis­(acetylacetonate) (TDBA) (75 wt % in isopropanol, Sigma-Aldrich) in 2-propanol (1:7 v/v) for deposition. Then, for the entire batches, the c-TiO_2_ layer was spin-coated at 5000 rpm for 30 s and annealed for 30 min at 500 °C. After cooling down the substrates to room temperature, diluted TiO_2_ paste purchased from the Great Cell Solar Company (18NRT, w/w = 1:3.5 in ethanol) was spin-coated on all three batches at 4000 rpm for 30 s, followed by an annealing process at 500 °C for 30 min and cooling down to room temperature. Then, for the second and third batches, the suspensions of the synthesized CeO_2_ rods and cubes in cyclohexane were spin-coated at 2000 rpm for 30 s, respectively. To understand the effect of thickness, the CeO_2_ layer was also spin-coated at 1000 and 3000 rpm for a separate batch of devices. Subsequently, these batches were annealed at 150 °C for 30 min and gradually cooled to room temperature.

**Table 1:** Configuration of the Fabricated Perovskite Devices

Type	C-TiO_2_	m-TiO_2_	CeO_2_ Rod	CeO_2_ Cube	Al_2_O_3_	Carbon	Perovskite
Device 1	present	present	N/A	N/A	present	present	present
Device 2	present	present	present	N/A	present	present	present
Device 3	present	present	N/A	present	present	present	present

Afterward, for all the batches, the Al_2_O_3_ mesoporous layer was spin-coated with the diluted Al_2_O_3_ paste (Sigma-Aldrich, 702129; v/v = 1:2 in isopropanol) at 3000 rpm for 30 s. Then, the substrates were annealed at 150 °C for 30 min and prepared for the carbon coating. The high-temperature carbon paste was prepared as per the previously published report with suitable modifications.^48^ The carbon layer screen was printed for all the substrates and annealed at 450 °C for 1 h. After cooling down to room temperature, the prepared perovskite precursor solution was drop-casted over the coated carbon layer and spin-coated for 20 s at 1500 rpm. The perovskite solution was prepared by using the ion-exchange method following our previous report: 0.191 g of methylammonium iodide (MAI), 0.553 g of lead iodide (PbI_2_), and 0.015 g of 5-aminovaleric acid iodide (5-AVAI) were mixed in 1 mL of γ-butyrolactone (GBL). This was followed by heating at 70 °C for 30 min and a filtration technique using a 0.2-μm PTFE filter to remove the sediments from the prepared perovskite precursor solution.^47^ At last, all of the batches underwent performance testing and characterization. The whole fabrication process and testing were performed under ambient conditions.

## 3 Characterization Techniques

The morphological analysis of the synthesized CeO_2_ rod and cube particles was performed by scanning electron microscopy (SEM, LEO 430i, Carl Zeiss). The particle shape analysis was conducted at a magnification of 50 K×, and the cross-sectional layer analysis at a magnification of 700×. The X-ray diffraction (XRD) of the material was executed by using X’pert pro-MPD XRD system from PANanalytical with Cu Kα1 radiation (λ = 1.5406 Å). The photovoltaic performance of the fabricated devices was evaluated with a Wacom AAA continuous solar simulator (model: WXS-210S-20, AM 1.5 G) under 1000 W/m^2^ illumination and an I–V tracer (EKO MP-160i). The EIS (electrochemical impedance spectroscopy) was executed through an AUTOLAB frequency analyzer setup equipped with both an FRA (frequency response analyzer) module and an AUTOLAB PGSTAT 10. The EIS measurement was carried out under dark conditions with an open-circuit voltage of 0.80 V and a frequency range of 1 MHz to 0.1 Hz. In order to fit the experimental data, the Z-view software (Version 3.4d, Scribner Associates, Inc., USA) was used. The IPCE (incident photon-to-current efficiency) and EQE (external quantum efficiency) were measured by using the BENTHAM PVE300 instrument equipped with a tungsten halogen lamp source, which provided a wavelength range of 350–750 nm. In order to analyze the diffused reflectance, a spectrophotometer (PerkinElmer Lambda 1050) study was conducted for three types of samples.^49–51^ The first sample was prepared by coating C-TiO_2_ and m-TiO_2_ on the FTO glass sheets, and the other two were prepared by coating a thin layer of the synthesized CeO_2_ nanostructures.

## 4 Results and Discussion

### 4.1 Chemical Composition and Structural Analysis

In order to determine the nature of the synthesized material, both chemical composition and microstructural studies were carried out. XRD studies were conducted to pinpoint the composition and crystalline nature of the materials. Since different crystalline materials possess unique diffraction patterns, Figure 2a represents the XRD pattern for the synthesized CeO_2_ nanostructures. The intensity of the diffraction peaks denotes the quantification of the relative amounts of the different phases in the synthesized sample. Figure 2a shows the XRD peaks near 28.80°, 33.50°, 47.60°, 56.70°, and 58.5° due to the (111), (200), (220), (311), (231), and (222) planes as the primary attributes of the sample. The peak planes resemble the crystalline phase of the CeO_2_ particles.^52–54^ The sharper peaks of the nanocubes indicated their higher crystallinity and larger crystallite size compared to those of the nanorods. Nanorods showed anisotropic peak broadening due to their elongated shape, while nanocubes exhibit more symmetric peak profiles. Similarly, the major peaks at ≈14°, 24.4°, 28.4°, and 31.8°, equivalent to the (110), (111), (201), and (211) planes, as displayed in Figure 2b, confirm the crystalline nature of the AVAI-MAPbI_3_ perovskite.^55^

**Figure 2:**
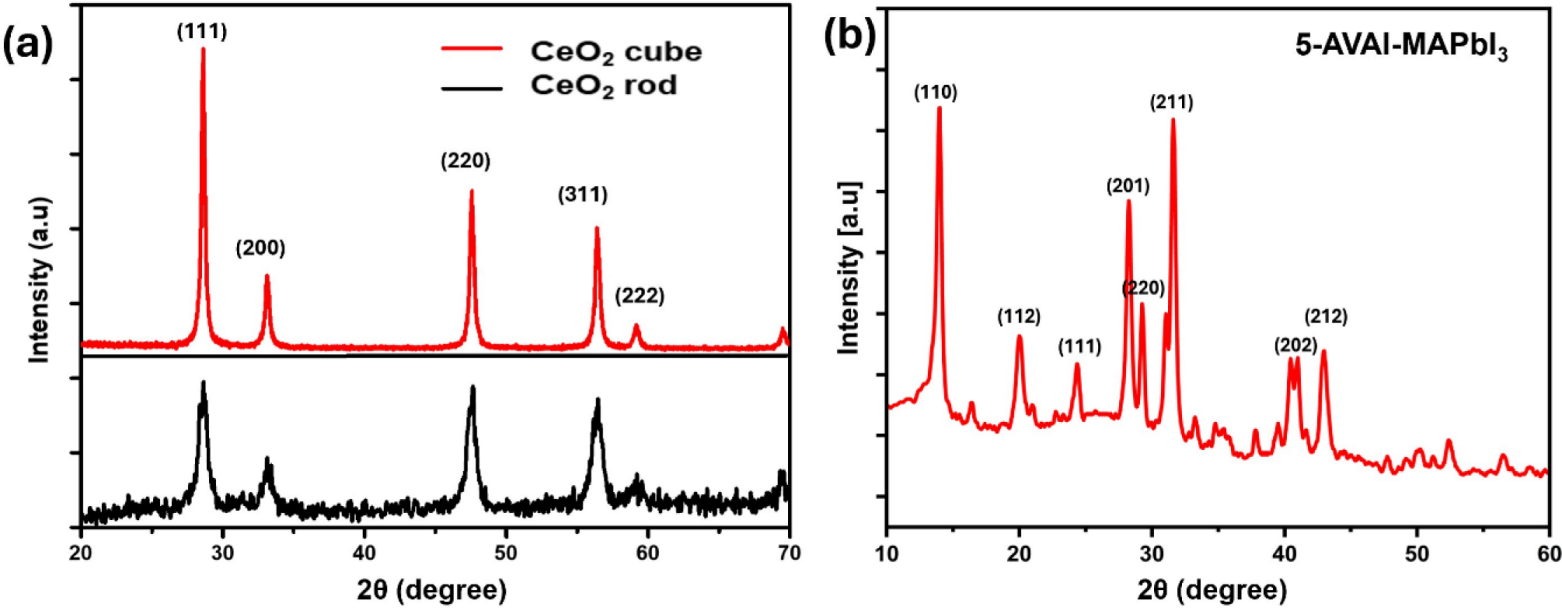
XRD patterns of the (a) CeO_2_ nanostructures and (b) 5-AVAI-MAPbI_3_, respectively.

The morphological variations of nanostructures usually have a critical effect on the reactivity, carrier mobility, and interface quality of thin-film devices.^56,57^ To understand the morphological difference of the synthesized nanostructures, SEM (scanning electron microscopy) was conducted. Figure 3a,b represents the rod and cubical structures, respectively, which resemble those observed in the previous study.^46^ In comparison with the well-defined facets and edges of the cube structure, the rod structure is elongated in one direction. Through the analysis of the microscopic images, it was found that the average diameter of the synthesized nanorods is nearly 40 nm, whereas the average length of the rod particles is ∼200 nm. On the other hand, the cube-like structure has an average crystallite size of ∼50 nm.

**Figure 3:**
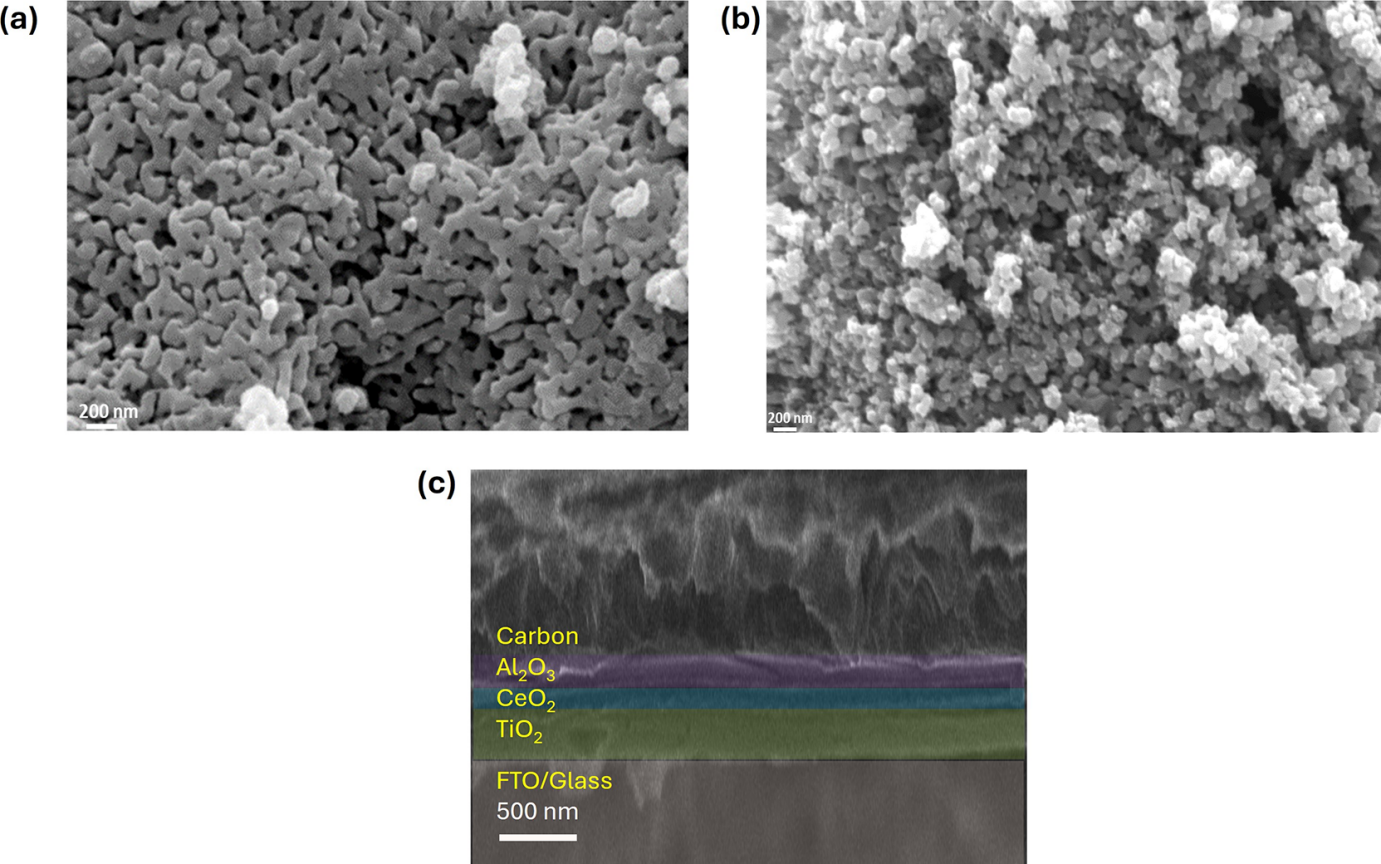
Microscopic characteristics of the materials: (a) SEM image for the CeO_2_ rod particles, (b) SEM image for the synthesized CeO_2_ cube particles, and (c) cross-sectional SEM image of a fabricated perovskite device showcasing different layers.

Moreover, Figure 3c shows a cross-sectional view of a fully fabricated perovskite device. Scaling and analysis dictate the thicknesses of the layers as ∼350, ∼160, and ∼240 nm for TiO_2_, CeO_2_, and Al_2_O_3_, respectively, on FTO-coated glass. Furthermore, as perovskite is infiltrated on top of the carbon layer, it is not expected that perovskite plates will be observed in the cross-sectional SEM image.

### 4.2 Spectroscopic Analysis

As the diffused reflectance study provides a better understanding of the light scattering effect through the perovskite layer, the spectrophotometer study of the three different samples was conducted, namely glass/TiO_2_, glass/TiO_2_/CeO_2_ rod, and glass/TiO_2_/CeO_2_ cube, as displayed in Figure 4a.^54^ Considering the fact that the higher diffused reflectance is directly proportional to the light penetration toward the perovskite sensitizer, the sample consisting of TiO_2_ and the CeO_2_ rod structure provides the most favorable light diffusion, followed by TiO_2_ and the combination of TiO_2_ and the cube structure.^54,58,59^ Even though the thickness of TiO_2_ remains unchanged for all of the samples, the changes in the diffused reflectance with respect to the two different CeO_2_ nanostructures indicate the effect of morphology and densely packed particles of thin films. Thus, the nanorods’ 1D structure enhances the light harvesting efficiency of the device, which, according to the literature, should decrease the electron transfer time from the perovskite to the photoanode, influencing an elongated recombination process.

**Figure 4:**
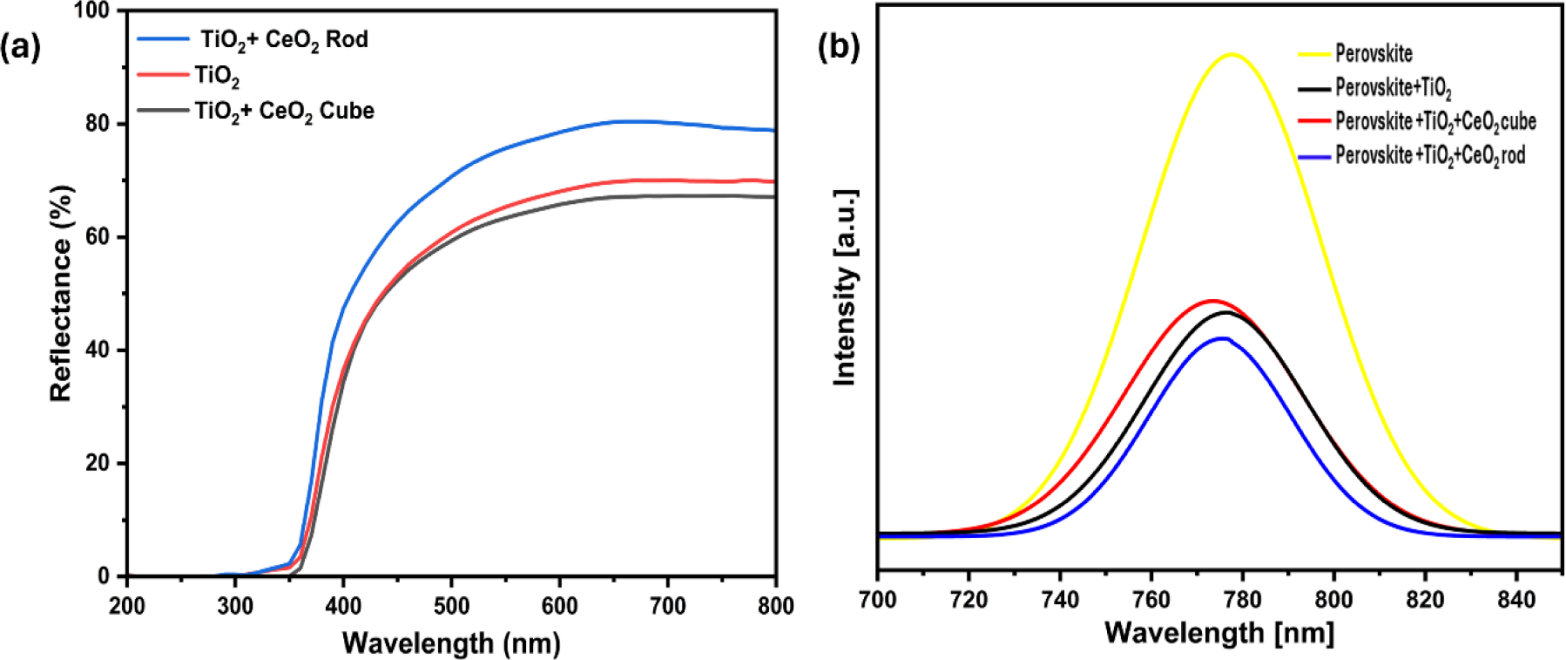
(a) Diffused reflectance analysis of the sample through the spectrophotometer study. (b) Steady-state photoluminescence pattern of the perovskite in the presence of the ETL.

To substantiate the findings, the photoluminescence of various ETLs and perovskite contacts was examined, which provided a fundamental understanding of charge transfer and interface quality, as shown in Figure 4b. At an excitation wavelength of 473 nm, a strong band-edge emission peak was observed in the samples, which was nearly at 775 nm. As expected, the most intense emission peak was observed for the perovskite film coated on top of the glass, whereas the quenching effect was exhibited by samples having additional transport layers. Due to the quenching effect, the films’ excited electrons will return to the ground state by energy transfer to the ETLs, which act as the quenching layer influencing charge transfer efficiency. In Figure 4b, the nanorod-structured CeO_2_/TiO_2_ as the ETL showed the most promising quenching effect with the frailest emission peak near 775 nm, which implies significant electronic energy release by energy transfer. However, previous studies indicate that photoluminescence behavior alone does not promise the suitable photovoltaic performance of devices due to other photoelectrical factors of the C-PSC.

Importantly, a significant shift in wavelength was detected for the CeO_2_ rod toward the blue region in the PL spectra, which authenticates the passivation of deep-level traps at grain boundaries, reducing the recombination centers and impacting higher charge transfer for the perovskite film.^60^ Similarly, reduced surface defects due to the diminished grain boundaries of nanorods are expected to play a key role in enhancing the electrical properties of the devices.

### 4.3 Photovoltaic Performance Analysis

In order to conduct the photovoltaic performance analysis, three batches of cells with the configuration mentioned in Table 1 were prepared, and the performance study was conducted. Additionally, the EQE (external quantum efficiency) and EIS (electrochemical impedance spectroscopy) were investigated to analyze photovoltaic behavior and the effect of morphology modulations on the performance. Table 2 represents the current density–voltage characteristics of different devices. In comparison to the results of the cells with the combined CeO_2_ cube nanostructure and only TiO_2_ ETLs, the cell configuration with the combined CeO_2_ rod/TiO_2_ ETL showed the best performance. For the rod structure, the champion cell achieved a maximum PCE of 12.38% with a *V*_oc_, *J*_sc_, and FF of 946.96 mV, 21.1 mA/cm^2^, and 0.620, respectively. Figure 5a exhibits the nature of the *J*–*V* curves of all three types of champion devices. From the high fill factor, it can be inferred that the TiO_2_ modified with the CeO_2_ nanorod reduces the loss of photoexcited carriers, suppressing surface electron recombination. Meanwhile, the increase in *J*_sc_ is mainly due to the high light-harvesting efficiency toward the photosensitizer. The results obtained from the photovoltaic performance study of all three batches align with the spectroscopic analysis findings.

**Table 2:** Photovoltaic Performance Characteristics of the C-PSC Fabricated with Three Configurations under 1 Sun AM 1.5G with a 0.25 cm^2^ Active Area

Device Type	*V*_oc_ (mV)	*J*_sc_ (mA/cm^2^)	Fill Factor (FF)	PCE (%)
**TiO_2_ + CeO_2_** **rod**	946.9	21.1	0.62	12.38
**TiO_2_ + CeO_2_** **cube**	945.4	19.0	0.51	9.16
**TiO_2_ only**	928.4	19.2	0.56	9.98

**Figure 5:**
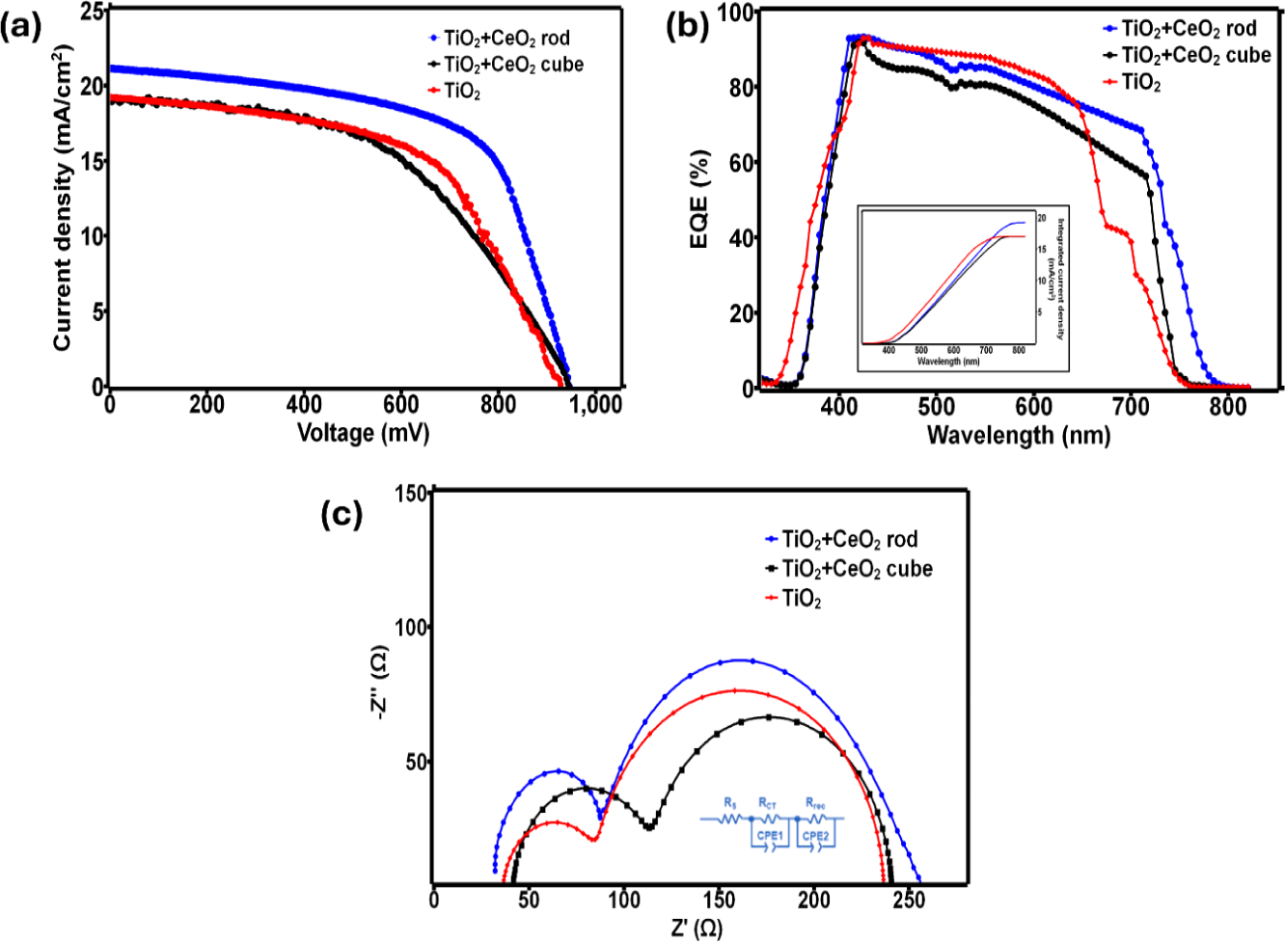
(a) Photovoltaic performance of the three devices with different configurations, (b) corresponding EQE representation of the devices (inset: integrated photocurrent density plot), and (c) EIS nature of the devices with the fitted electrical circuit diagram (inset: circuit diagram).

To understand the effect of fabrication engineering, photovoltaic performance was observed for CeO_2_-based devices with varying spin-coating speeds, as detailed in Table 3. The observations suggested that 2000 rpm achieved the optimum thickness suitable for devices with nanorod and nanocube morphologies.

**Table 3:** Photovoltaic Performance of the Champion CeO_2_ Nanostructure Devices at Different Spin-Coating Speeds under 1 Sun AM 1.5, Having an Active Area of 0.25 cm^2^

Device	*J*_sc_ (mA/cm^2^)	*V*_oc_ (mV)	Fill Factor (FF)	PCE (%)
**CeO_2_ rod 1000 rpm**	20.4	925.2	0.61	11.5
**CeO_2_ rod 3000 rpm**	20.8	932.6	0.615	11.9
**CeO_2_ cube 1000 rpm**	18.6	924.8	0.503	8.65
**CeO_2_ cube 3000 rpm**	18.9	934.4	0.506	8.9

Next, the EQE (external quantum efficiency) analysis provides insight into the spectral performance of the C-PSC with three configurations across visible wavelengths of light.^58,61,62^ As shown in Figure 5b, the cell configuration with the CeO_2_ rod structure demonstrates the highest EQE of ∼92% compared to those of the other two configurations. This indicates that the rod surface morphology reduces light trapping and enhances scattering effects within the device.^63–65^ The integrated photocurrent density was calculated from the IPCE measurement (inset, Figure 5b) and found to be around 18.5 mA/cm^2^, 16.7 mA/cm^2^, and 16.5 mA/cm^2^ for CeO_2_ rod/TiO_2_, only TiO_2_, and CeO_2_ cube/TiO_2_ devices, respectively. The effect of optical losses caused by transmission and reflection produced slightly reduced values of integrated photocurrent densities compared to the values obtained via *J*–*V* characterization. Noticeably, strong IPCE coverage in the range of ∼500 to 780 nm for CeO_2_ rod-based devices demonstrates significant improvement in interface quality and proficient charge carrier transport compared to the commercial TiO_2_-based ETL, certifying the surface defect reduction.^38^

The EIS analysis implicates the rationale behind the changes in the C-PSC performance metrics such as *V*_oc_, *J*_sc_, and FF of each cell. The representation of EIS reveals information about the transport process and charge carrier recombination of the perovskite layers at the interfaces or the adjacent layers.^66–68^ Primarily, this provides insight into the charge extraction process from the perovskite to the ETL and the perovskite to counter electrode interfaces.^67–69^ Figure 5c indicates the impedance response of three C-PSCs with different combinations. The *R*_s_ (series resistance) indicates the impedance response of the cell between the two electrodes, which are FTO and carbon, and displays that the C-PSC configuration with a rod structure provides a lower *R*_s_ compared to the cube and the normal titania.^54,66,70–73^ The *R*_CT_ represents the resistance between the carbon and perovskite layers. The *R*_rec_ (charge recombination resistance) in the equivalent circuit diagram quantifies the resistance between the perovskite and ETLs.^54,66–68,70–72^ Commonly, a lower *R*_CT_ value favors the enhanced photocurrent density of the C-PSC due to the superior charge collection ability between the perovskite and the counter electrode.^67,70,71^ The differences in the *R*_CT_ values for the three configurations of C-PSCs are clearly visible in Figure 5c and Table 4.

**Table 4:** EIS Parameters of the Champion C-PSC Devices with and without CeO_2_

Device Type	*R*_s_ (Ω)	*R*_CT_ (Ω)	*R*_rec_ (Ω)
**TiO_2_ + CeO_2_** **rod**	32.1	57.1	182.2
**TiO_2_ + CeO_2_** **cube**	42.6	75.2	130.3
**TiO_2_ only**	36.1	49.7	151.1

Since the particle shape and size affect the photovoltaic performance of the C-PSCs, the grain boundary effect on the nanoparticle shape of CeO_2_ with the cube structure hinders the enhancement of the photovoltaic performance of the cell.^70,74^ While the grain boundaries can act as recombination centers for the charge carriers, this increases the recombination rates and reduces the charge carrier lifetime.^37,74–77^ The lower *R*_rec_ value in the C-PSC with the cube structure mirrors the higher recombination process at the ETL and perovskite interface, which gradually reduces the photovoltaic performance of the cells.^66,67,71^ On the other hand, the higher *R*_rec_ value of the cell with the rod structure indicates a lower recombination rate, which gives an upper hand to the usage of rod-structured CeO_2_ in the C-PSC. Overall, the lower *R*_CT_ and high *R*_rec_ values of the C-PSC with the CeO_2_ rod make it a better candidate for the HTL-free carbon-based perovskite device compared to only commercial TiO_2_ devices.

This highlights the need to detail the effects of nanostructures in perovskite photovoltaics, regarding enhanced light harvesting, electron transfer, and trap passivation. Nanorods offer distinct advantages over nanocubes in light harvesting and electron transfer in perovskite solar cells due to their unique one-dimensional structure, which results in a higher aspect ratio, allowing them to scatter and trap light more effectively compared to nanocubes. This increases the optical path length, leading to better light absorption by the perovskite layer.^78,79^ The one-dimensional structure of nanorods provides a direct pathway for electron transport, reducing recombination losses. This anisotropic charge transport is more efficient than the random pathways in cube-based systems, leading to faster and more reliable electron transfer from the perovskite to the photoanode.^78^ Further, nanorods minimize electron trapping sites and recombination rates due to their well-aligned structure, which is less prone to defects compared to the aggregated nature of nanoparticles.^79^ Moreover, nanorods create a more uniform and intimate contact with the perovskite layer, enhancing charge injection and overall device performance.^80^ Consequently, rod-type nanostructures have distinct advantages in passivating perovskite traps compared to nanocubes, largely due to their elongated shape, anisotropic properties, and unique interactions with perovskite materials. The rods can integrate more effectively within the perovskite matrix due to their anisotropic structure. Their directional orientation supports better alignment with perovskite grains, optimizing the suppression of defect states.^81^ Unlike nanocubes, rod-type nanostructures facilitate directional charge transport through their elongated geometry. This reduces charge carrier losses and enhances overall device efficiency.^82^ Furthermore, the elongated shape allows rods to interact with defects that might be less accessible to smaller nanocubes. This leads to more thorough trap passivation and stabilization.^83^

Similarly, grain boundaries in nanorods and nanocubes have distinct effects on the electron transport layer (ETL) in perovskite solar cells due to their differing morphologies and structural characteristics. Grain boundaries in nanorods are aligned along their elongated structure, which facilitates the directional charge transport. This reduces recombination losses and enhances the efficacy of the ETL.^84^ Also, 1D nanorods can penetrate deeper into the perovskite layer, addressing defects at grain boundaries more effectively. This leads to improved trap passivation and stability. On the other hand, grain boundaries in nanocubes are distributed isotropically, which can lead to uniform but less directional charge transport. This may result in higher recombination losses compared to nanorods.^85^ Ultimately, the reason for photovoltaic performance variation due to the morphology alteration is clear from the diffuse reflectance, PL, EQE, and EIS characterization.

Finally, the repeatability test was performed (Figure 6a), and the stability of the devices was observed under ambient conditions, monitored for 500 h, with photovoltaic characterization performed every 100 h (Figure 6b). The repeatability test for a batch of 6 devices produced average efficiencies of 11.0%, 8.3%, and 8.9% for the CeO_2_ rod, CeO_2_ cube, and pristine TiO_2_-type devices, respectively. On the other hand, only 15% efficiency loss was observed for the CeO_2_ devices during stability testing, whereas ∼20% PCE loss was observed for TiO_2_ devices. Some studies have described the extraction of electrons from I^–^ by TiO_2_ and oxidizing I^–^ to iodine in the presence of light as a deforming factor of the perovskite crystal structure; as a result, slightly low stability was observed for commercial TiO_2_-based devices.^86,87^ However, the presence of CeO_2_ neutralizes this effect to a great extent, demonstrating a noteworthy stability improvement in the respective devices. The aim of this work is to discuss the morphological effect of nanomaterials and device engineering combinations, and we believe this study can provide further advancements in the field of perovskite photovoltaics.

**Figure 6:**
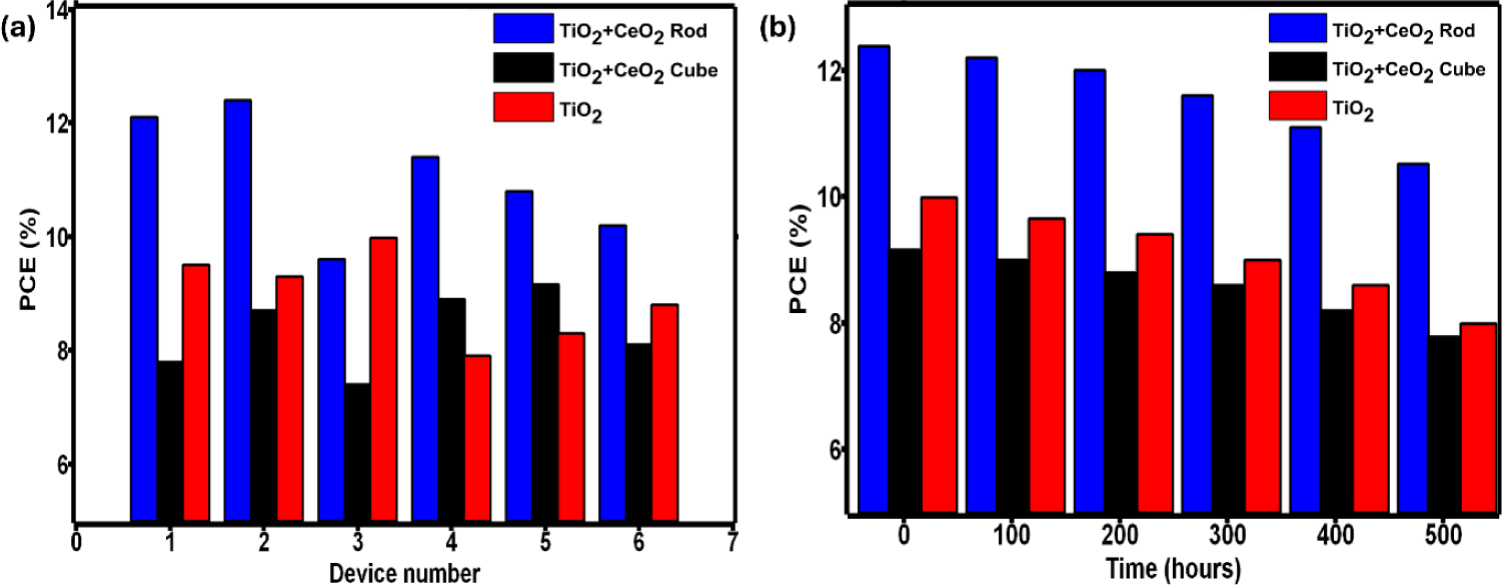
(a) Repeatability test for a batch of 6 devices from each type and (b) stability data (PCE vs time) of different types of devices after 500 h while maintained under ambient conditions.

## 5 Conclusion

In conclusion, two different CeO_2_ nanostructures, rod and cube, were synthesized and added over the TiO_2_ layer of the C-PSC. Integrally, the combination of TiO_2_ and morphology-modulated CeO_2_ acts as the ETL for the C-PSC, and the major part of the analysis was carried out by comparing the combined ETL and the normal ETL effects on the C-PSC. The device engineering showcased due to the CeO_2_ nanostructures transformed the electron transfer property, light-harvesting efficiency, and the photovoltaic performance of the C-PSCs significantly. The cell configuration with the rod nanostructure indicated the highest PCE performance of 12.38% over 9.16% and 9.98% of the cube structure and the normal TiO_2_, respectively. This suggests that the morphology modulation of the nanoparticles and allied grain boundary trap passivation contributes significantly to the performance of the C-PSCs. Moreover, the spectrophotometer and EIS analysis provided a better understanding of the effect of charge recombination rates on morphology modulation. The variations in the diffused reflectance denote the correlation effect of the light-scattering effect of the morphology-modulated CeO_2_ and the combined effect with the TiO_2_ particles toward better light harvesting. The results from the experimentation establish a foundation for the significant importance of morphology-modulated nanostructures and fabrication engineering of the devices in the field of HTL-free C-PSCs. From this work, we anticipate that the results may open up pathways for future advancements in the morphology optimization of nanomaterials for C-PSC application and facilitate their feasible integration into real-world applications.

## Data Availability

The dataset is available on request.
